# Interventions for Adolescent Mental Health: An Overview of Systematic Reviews

**DOI:** 10.1016/j.jadohealth.2016.06.020

**Published:** 2016-10

**Authors:** Jai K. Das, Rehana A. Salam, Zohra S. Lassi, Marium Naveed Khan, Wajeeha Mahmood, Vikram Patel, Zulfiqar A. Bhutta

**Affiliations:** a Division of Women and Child Health, Aga Khan University, Karachi, Pakistan; b Robinson Research Institute, University of Adelaide, Adelaide, Australia; c Ziauddin University, Karachi, Pakistan; d London School of Hygiene & Tropical Medicine, London, United Kingdom; e Public Health Foundation of India, New Delhi, India; f Sangath, Goa, India; g Centre for Global Child Health, The Hospital for Sick Children, Toronto, Canada; h Center of Excellence in Women and Child Health, The Aga Khan University, Karachi, Pakistan

**Keywords:** Adolescent health, Mental health, Suicide, Depression, Anxiety, Eating disorders

## Abstract

Many mental health disorders emerge in late childhood and early adolescence and contribute to the burden of these disorders among young people and later in life. We systematically reviewed literature published up to December 2015 to identify systematic reviews on mental health interventions in adolescent population. A total of 38 systematic reviews were included. We classified the included reviews into the following categories for reporting the findings: school-based interventions (n = 12); community-based interventions (n = 6); digital platforms (n = 8); and individual-/family-based interventions (n = 12). Evidence from school-based interventions suggests that targeted group-based interventions and cognitive behavioral therapy are effective in reducing depressive symptoms (standard mean difference [SMD]: −.16; 95% confidence interval [CI]: −.26 to −.05) and anxiety (SMD: −.33; 95% CI: −.59 to −.06). School-based suicide prevention programs suggest that classroom-based didactic and experiential programs increase short-term knowledge of suicide (SMD: 1.51; 95% CI: .57–2.45) and knowledge of suicide prevention (SMD: .72; 95% CI: .36–1.07) with no evidence of an effect on suicide-related attitudes or behaviors. Community-based creative activities have some positive effect on behavioral changes, self-confidence, self-esteem, levels of knowledge, and physical activity. Evidence from digital platforms supports Internet-based prevention and treatment programs for anxiety and depression; however, more extensive and rigorous research is warranted to further establish the conditions. Among individual- and family-based interventions, interventions focusing on eating attitudes and behaviors show no impact on body mass index (SMD: −.10; 95% CI: −.45 to .25); Eating Attitude Test (SMD: .01; 95% CI: −.13 to .15); and bulimia (SMD: −.03; 95% CI: −.16 to .10). Exercise is found to be effective in improving self-esteem (SMD: .49; 95% CI: .16–.81) and reducing depression score (SMD: −.66; 95% CI: −1.25 to −.08) with no impact on anxiety scores. Cognitive behavioral therapy compared to waitlist is effective in reducing remission (odds ratio: 7.85; 95% CI: 5.31–11.6). Psychological therapy when compared to antidepressants have comparable effect on remission, dropouts, and depression symptoms. The studies evaluating mental health interventions among adolescents were reported to be very heterogeneous, statistically, in their populations, interventions, and outcomes; hence, meta-analysis could not be conducted in most of the included reviews. Future trials should also focus on standardized interventions and outcomes for synthesizing the exiting body of knowledge. There is a need to report differential effects for gender, age groups, socioeconomic status, and geographic settings since the impact of mental health interventions might vary according to various contextual factors.

Adolescence is a period for the onset of behaviors and conditions that not only affect health at that time but also lead to adulthood disorders. Unhealthy behaviors such as smoking, drinking, and illicit drug use often begin during adolescence and are closely related to increased morbidity and mortality and represent major public health challenges [1]. Many mental health disorders emerge in mid- to late adolescence and contribute to the existing burden of disease among young people and in later life [2]. More than 50% of adult mental disorders have their onset before the age of 18 years [3], [4]. Poor mental health has been associated with teenage pregnancy, HIV/AIDS, other sexually transmitted diseases, domestic violence, child abuse, motor vehicle crashes, physical fights, crime, homicide, and suicide [2]. Globally, neuropsychiatric disorders are the leading cause of years lost because of disability among 10- to 24-year-olds, accounting for 45% of years lost because of disabilities [5]. The overall prevalence of depression in adolescents is around 6% and that for children (younger than 13 years) is 3% [6]. Major depressive disorder (MDD) is one of the leading causes of disability, morbidity, and mortality and is a major risk factor for suicide [7]. MDD also puts adolescents and young adults at a greater risk for suicide as they are seven times more likely to complete suicide than those without MDD [8]. Suicide itself accounts for 9.1% of deaths in 15- to 19-year age group and ranks as the third major cause of mortality in this age group, preceded only by accidents and assault [9].

Given the prevailing burden and impact of mental health disorders in children and adolescents, it is essential that effective interventions are identified and implemented. This article is part of a series of reviews conducted to evaluate the effectiveness of potential interventions for adolescent health and well-being. Detailed framework, methodology, and other potential interventions have been discussed in separate articles [10], [11], [12], [13], [14], [15], [16]. Our conceptual framework depicts the individual and general risk factors through the life cycle perspective that can have implications at any stage of the life cycle [10]. We also acknowledge the fact that mental health interventions take a life course perspective and that interventions earlier in life can have impacts in adolescence; however, the focus of our review is to evaluate potential mental health interventions targeted toward adolescents and youth only. With this focus, we aimed to systematically review the effectiveness of interventions to prevent and manage mental health disorders among adolescents and youth.

## Methods

We systematically reviewed literature published up to December 2015, to identify systematic reviews on interventions to prevent and manage mental health disorders in adolescent population. For the purpose of this review, the adolescent population was defined as aged 11–19 years; however, since many studies targeted youth (aged 15–24 years) along with adolescents, exceptions were made to include reviews targeting adolescents and youth. We did not apply any limitations on the start search date or geographical settings. We considered all available published systematic reviews on the interventions to prevent and treat adolescent mental health disorders. A broad search strategy was used that included a combination of appropriate keywords, medical subject heading, and free text terms; the search was conducted in the Cochrane Library, and PubMed. The abstracts (and the full sources where abstracts are not available) were screened by two abstractors to identify systematic reviews adhering to our objectives. Any disagreements on selection of reviews between these two primary abstractors were resolved by the third reviewer. After retrieval of the full texts of all the reviews that met the inclusion/exclusion criteria, data from each review were extracted independently into a standardized form. Information was extracted on (1) the characteristics of included studies; (2) description of methods, participants, interventions, outcomes; (3) measurement of treatment effects; (4) methodological issues; and (5) risk of bias tool. We extracted pooled effect size for outcomes reported by the review authors with 95% confidence intervals (CIs). We assessed and reported the quality of included reviews using the 11-point assessment of the methodological quality of systematic reviews criteria (AMSTAR) [17]. We excluded nonsystematic reviews, systematic reviews focusing on preventive and therapeutic mental health interventions targeting population other than adolescents and youth, and reviews not reporting outcomes related to mental health (Table 1).

**Figure 1:**
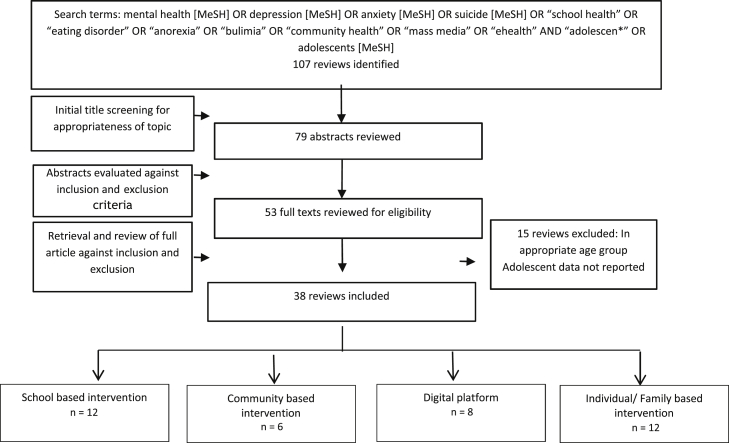
Search flow diagram. MeSH = Medical Subject Heading.

Figure 1 describes the search flow. Our search identified 107 potentially relevant review titles. Further evaluation of the abstracts and full texts resulted in the inclusion of 38 eligible reviews. We classified the included reviews into the following categories for reporting the findings:

• School-based interventions (n = 12)
• Community-based interventions (n = 6)
• Digital platforms (n = 8)
• Individual-/family-based interventions (n = 12)

Table 2 describes the characteristics of the included reviews while Table 3 provides the summary estimates for all the interventions.

## Results

### School-based interventions

We found a total of 12 reviews reporting school-based interventions for adolescent mental health, of which one review performed meta-analysis. AMSTAR rating ranged between 5 and 11 with a median score of 7.5. Five of the included reviews focused on school-based mental health promotion interventions; three reviews evaluated school-based programs for prevention and early intervention for existing mental health conditions while four reviews evaluated school-based programs for suicide prevention. A review on school mental health promotion programs based on the findings from 15 studies suggests that an approach focusing on mental health promotion rather than on mental illness prevention is effective in promoting adolescent and youth mental health [18]. However, study populations were limited, and studies either lack clarity regarding who implemented interventions or lack theoretical foundations, process evaluations, or youth viewpoints [18]. Meta-analysis was not conducted due to variations in interventions and outcomes. Another review reported from 27 studies that school-based preventive health care is popular with young people and provides important mental health services [19]. However, meta-analysis was not done due to study quality. Findings from a review based on 16 studies focusing on targeted group-based interventions delivered in school settings suggest that nurture groups (short-term, focused intervention which addresses barriers to learning arising from social, emotional, or behavioral difficulties in an inclusive, supportive manner) have an immediate positive impact on the social and emotional well-being of young people [20]. Due to heterogeneity of design, it was not possible to conduct a meta-analysis, and the studies were examined for effectiveness qualitatively. A review evaluating solution-focused brief therapy in schools has suggested mixed results with some promise in working with students in school settings, specifically for reducing the intensity of students' negative feelings, managing conduct problems, and externalizing behavioral problems [21]. These findings are based on seven studies while meta-analysis could not be conducted. School-based mental health interventions specifically focusing on low- and middle-income countries (LMICs) suggest that the majority of the school-based life skills and resilience programs indicated positive effects on students' self-esteem, motivation, and self-efficacy. However, there were mixed results, including differential effects for gender and age groups [19], and effect estimates could not be pooled. A systematic review on the effectiveness of school nurse implemented mental health screening for adolescents in schools did not find any evidence of existing screening tools to detect mental ill health among adolescents in schools [22].

A systematic review of 28 school-based prevention and early intervention programs for depression has shown some support for the implementation of depression prevention and early intervention programs in schools [23]. Most of these programs were based on cognitive behavioral therapy (CBT) and delivered by a mental health professional or graduate student over 8–12 sessions. Indicated programs, which targeted students exhibiting elevated levels of depression, were found to be the most effective in reducing depressive symptoms with effect sizes ranging from .21 to 1.40. Meta-analysis was not conducted. It was found that CBT delivered to young people in secondary schools can reduce the symptoms of depression (standard mean difference [SMD]: −.16; 95% CI: −.26 to −.05) and anxiety (SMD: −.33; 95% CI: −.59 to −.06) [24]. School-based therapeutic mental health programs specifically targeting adolescents with existing mental health disorders in LMICs suggested negative effects for programs that targeted externalizing problems and were delivered selectively to youth with existing problems. Distinctive characteristics of low-income, urban schools, and nonschool environments were emphasized as potential explanations for the findings [25].

School-based suicide prevention programs focused on awareness/education curricula, screening, gatekeeper, peer leadership, and skills training [26], [27]. Interventions for primary prevention of suicide in university and other postsecondary educational settings suggest that classroom-based didactic and experiential programs increased short-term knowledge of suicide (SMD: 1.51; 95% CI: .57–2.45) and knowledge of suicide prevention (SMD: .72; 95% CI: .36–1.07) with no evidence of an effect on participant's suicide-related attitudes or behaviors; however, these findings are limited by the overall low quality [28]. Promising interventions that need further research include school-based prevention programs with a skills training component, individual CBT interventions, interpersonal psychotherapy, and attachment-based family therapy [26], [27]. A systematic review evaluating suicide prevention programs targeting indigenous youth (aboriginals) suggested that more controlled study designs using planned evaluations and valid outcome measures are needed in research on indigenous youth suicide prevention [29].

### Community-based interventions

We report findings from six systematic reviews evaluating various community-based interventions targeting adolescents and youth; meta-analysis was conducted in two reviews. AMSTAR ratings ranged between 4 and 7 with a median score of 5. Evidence from 20 studies evaluating community-based creative activities (including music, dance, singing, drama, and visual arts) suggests some positive effect on behavioral changes, self-confidence, self-esteem, levels of knowledge, and physical activity [30]. The interventions used in the studies were diverse, and the research was heterogeneous, and hence overall synthesis of the results was not attempted. Another review based on 15 studies on community-based parent training and social skills training for preventing depression suggested significant reductions in symptom and/or diagnostic measures at follow-up [31]. However, meta-analysis was not conducted. Evidence from a review evaluating primary prevention mental health programs for adolescents suggests that individually focused mental health promotion efforts and attempts to help negotiate stressful transitions yield significant mean effects on reducing problems and increasing competencies [32]. Evidence from community-based mental health delivery programs specifically targeting mental health promotion of young people in LMICs suggests positive impacts on mental health outcomes; however, pooled analysis could not be conducted [19]. Another review evaluating community-based mental health and behavioral programs for low-income urban youth suggested that person-only interventions had a nonsignificant impact on improving mental health (measured by an aggregate outcome measure; SMD: .03; 95% CI: −.19 to .25) while person plus environmental interventions (SMD: .27; 95% CI: .16–.37) and environment-only interventions had a significant positive impact (SMD: .38; 95% CI: .15–.60) [33]. One review reporting the impact of treatment of adolescent mental health disorders in primary care settings suggests some preliminary evidence that treatments by specialist staff working in primary care were effective, although quality of included studies was variable. Meta-analysis could not be conducted. Some educational interventions showed potential for increasing skills and confidence of primary care staff, but controlled evaluations were rare, and few studies reported the actual change in professional behavior or patient health outcomes [34].

### Digital platforms for mental health interventions

We report findings from eight systematic reviews evaluating impact of digital platforms for mental health disorders. None of the included reviews conducted meta-analysis. AMSTAR rating ranged between 4 and 11 with a median score of 9. A review evaluating the impact of mass media interventions from two studies suggests an impact ranging from SMD −.85 to −.17 on discrimination while the impact on prejudice ranged between SMD −2.94 and 2.40. The studies were very heterogeneous, statistically, in their populations, interventions, and outcomes, and hence meta-analysis could not be conducted [35]. Evidence pertaining to mass media suggests that mass media–based behavioral treatments have a moderate effect while computerized CBT for mental health suggests that such interventions are cost-effective and often cheaper than usual care [36], [37]. Another review evaluating online youth mental health promotion and prevention interventions indicates that there is some evidence that skills-based interventions presented in a module-based format can have a significant impact on adolescent mental health; however, an insufficient number of studies limit this finding. The results from online interventions indicate significant positive effect of computerized CBT on adolescents' and emerging adults' anxiety and depression symptoms [38]. These findings are based on 20 studies; however, meta-analysis could not be conducted in this review due to heterogeneity in studies. Evidence from four Internet-based prevention and treatment programs for anxiety and depression suggests early support for the effectiveness; however, more extensive and rigorous research is warranted to further establish the conditions through which effectiveness is enhanced, as well as to develop additional programs to address gaps in the field [39]. Three reviews evaluating the acceptability and feasibility of mental health resources among youth suggested that young people regularly use and are generally satisfied with online mental health resources [40], [41], [42].

### Individual-/family-based interventions

We included 12 systematic reviews focusing on individual- or family-based interventions, of which 10 reviews performed meta-analysis. AMSTAR rating ranged between 6 and 11 with a median score of 11. One review focused on interventions for eating disorders; four reviews focused on physical activity and exercise interventions; six reviews focused on CBT, psychotherapy, behavioral, and pharmacological interventions for anxiety and depression; while two reviews focused on home-based multisystemic interventions.

A systematic review on the effectiveness of eating disorder programs for adolescents focused on eating disorder awareness, healthy eating attitudes and behaviors, media literacy and advocacy skills, and promoting self-esteem [43]. All included studies were conducted in high-income countries (HICs). Interventions focusing on eating attitudes and behaviors showed no impact on body mass index at 12- to 14-month follow-up (SMD: −.10; 95% CI: −.45 to .25), Eating Attitude Test at 6- to 12-month follow-up (SMD: .01; 95% CI: −.13 to .15), and bulimia at 12- to 14-month follow-up (SMD: −.03; 95% CI: −.16 to .10). Combined data from two eating disorder prevention programs based on a media literacy and advocacy approach showed a significant reduction in the internalization or acceptance of societal ideals relating to appearance at a 3- to 6-month follow-up (SMD: −.28; 95% CI: −.51 to −.05). Two studies focusing on self-esteem approach showed no impact on close friendships (SMD: −.01; 95% CI: −.09 to .06) and social acceptance (SMD: −.03; 95% CI: −.10, .04) at 3-month follow-up. There is not enough evidence to suggest any harm from any of the prevention programs included in the review.

Four systematic reviews evaluated the impact of exercise and physical activity on mental health outcomes among adolescents and youth. Exercise alone was evaluated in eight studies showing significant impact on self-esteem (SMD: .49; 95% CI: .16–.81). Exercise as a part of other comprehensive interventions was evaluated in four studies and showed a significant improvement in self-esteem (SMD: .51; 95% CI: .15–.88). However, these conclusions are based on several small number of trials reporting poolable data with lack of long-term follow-up data [44]. Another review reporting the effects of physical activity programs (including outdoor adventure, sport and skill-based and physical fitness program) included 15 studies. Due to small number of studies and large heterogeneity in terms of study length, sample size, assessment of outcomes, and participants, meta-analysis was not conducted. Some studies suggested positive impacts on social and emotional well-being; however, due to mixed findings and the high risk of bias, the efficacy of physical activity programs could not be concluded [45]. Evidence on the use of exercise for depression compared to no treatment suggests significant impact in reducing depression from 35 trials (SMD: −.62; 95% CI: −.81 to −.42) while there was no impact on dropouts (relative risk [RR]: 1.00; 95% CI: .97–1.04). Exercise when compared to psychological therapy and pharmacological treatment found no significant difference on depression (SMD −.03; 95% CI: −.32 to .26 and SMD: −.11; 95% CI: −.34 to .12, respectively) [46]. Vigorous exercise when compared to no intervention led to reduced depression score (SMD: −.66; 95% CI: −1.25, −.08) with no impact on anxiety scores (SMD: −.48; 95% CI: −.97, .01) while vigorous exercise when compared to low intensity exercise and psychosocial interventions showed comparable results. However, the small number of studies and the clinical diversity of participants, interventions, and methods of measurement limit the ability to draw conclusions [47].

Six systematic reviews reported findings on interventions for anxiety and depression among adolescents and youth. A review on the effectiveness of CBT for anxiety disorders included 41 studies. CBT compared to waitlist was effective in reducing remission (odds ratio [OR]: 7.85; 95% CI: 5.31–11.6). There was nonsignificant impact on participants lost to follow-up (OR: .93; 95% CI: .58–1.51) [48]. A review evaluating the impact of psychological therapies and antidepressant medication, alone and in combination, for the treatment of depressive disorder for adolescents included 11 studies. Findings suggest that psychological therapy when compared to antidepressants had comparable effect on remission (OR: .62; 95% CI: .28–1.35), dropouts (OR: .61; 95% CI: .11–3.28), and depression symptoms (SMD: .16; 95% CI: −.69 to 1.01) while psychological therapy significantly reduced suicidal ideation (SMD: −3.12, 95% CI: −5.91 to −.33) when compared to antidepressant. Combination therapy was also found to be comparable to antidepressant medications for remission (OR: 1.50; 95% CI: .99–2.27), dropouts (OR: .84; 95% CI: .51–1.39), suicidal ideation (OR: .75; 95% CI: .26–2.16), depression symptoms (SMD: −.27; 95% CI: −4.95 to 4.41), and functioning (SMD: .09; 95% CI: −.11 to .28). Combination therapy was also found to be comparable to psychological therapy for remission (OR: 1.61; 95% CI: .38–6.90), dropouts (OR: 1.23; 95% CI: .12–12.71), suicidal ideation (SMD: .60; 95% CI: −2.25 to 3.45), and depression symptoms (SMD: −.28; 95% CI: −1.41 to .84). Psychological therapy when compared to combination therapy was effective in reducing remission (OR: 2.15; 95% CI: 1.15–4.02). Combination therapy significantly reduced depression symptoms (SMD: −.52; 95% CI: −.78 to −.26) compared to psychological therapy plus placebo [49]. Another review evaluating the impact of interventions for relapse and recurrence of depressive disorders included nine trials. Findings suggest reduction in number of relapsed recurred (OR: .34; 95% CI: .18–.64) with no impact on suicide-related behaviors (OR: 1.02; 95% CI: .14–7.39) and dropouts (1.02; 95% CI: .38–2.79) [50]. However, there is considerable diversity in the design of trials, making it difficult to compare outcomes across studies [50]. Behavioral therapy when compared to all other psychological therapies is reported to be equally effective for depression response (RR: .97; 95% CI: .86–1.09); remission (RR: .91; 95% CI: .8–1.04); response at follow-up (RR: .77; 95% CI: .59–1.01); depression severity (SMD: −.03; 95% CI: −.2–.15); and dropout (RR: 1.02; 95% CI: .65–1.61) [51]. Another review evaluating the performance of evidence-based youth psychotherapies compared with usual clinical care suggests that psychotherapies outperform usual care (SMD: .31; 95% CI: .16–.44), but the advantage is modest and moderated by youth, location, and assessment characteristics [52].

Evidence suggests that home-based multisystemic therapy resulted in improved externalizing symptoms, and they spent fewer days out-of-school and out-of-home placement. Intensive home-based crisis intervention using the “Homebuilders” model (components include relationship building, reframing problems, anger management, communication, setting treatment goals, and CBT) did not show any impact when compared to routine inpatient care [53]. Day therapy programs for adolescents with mental health disorders (including anxiety disorders, social phobia, and behavioral issues) suggest that it may be an effective intervention for adolescents with mental health disorders. A multimodal and multidisciplinary group-based treatment approach has shown to be most effective, and participants could benefit from the involvement of at least one health professional from a psychology or psychiatric background. Further high-level, high-quality research using standardized outcome measures is required to support these findings and determine key parameters, such as an optimal frequency and duration for day therapy programs [54].

## Discussion

We report findings from a total of 38 systematic reviews with an AMSTAR rating ranging between 7 and 11 and a median score of 8. Evidence from school-based interventions suggests that targeted group-based interventions and CBT were found to be effective in reducing depressive symptoms and anxiety. School-based suicide prevention programs suggest that classroom-based didactic and experiential programs increased short-term knowledge of suicide and knowledge of suicide prevention with no evidence of an effect on suicide-related attitudes or behaviors. Community-based creative activities had some positive effect on behavioral changes, self-confidence, self-esteem, levels of knowledge, and physical activity. Evidence from digital platforms supports Internet-based prevention and treatment programs for anxiety and depression; however, more extensive and rigorous research is warranted to further establish the conditions. Among individual- and family-based interventions, interventions focusing on eating attitudes and behaviors showed no impact on body mass index, Eating Attitude Test, and bulimia. Exercise was found to be effective in improving self-esteem and reduced depression score with no impact on anxiety scores. CBT compared to waitlist was effective in reducing remission. Psychological therapy when compared to antidepressants had comparable effect on remission, dropouts, and depression symptoms. Most of the evidence is from HICs, limiting the generalizability of the findings for LMICs. Meta-analysis could not be conducted in many of the included reviews due to heterogeneity in their populations, interventions, and outcomes.

One of the limitations of our review was that the scope of our review was limited to interventions targeting adolescents and youth only; however, mental health interventions take a life course perspective. Mental health disorders are linked in different ways and levels, exerting a dimensional effect between environmental, genetic factors and other biological mechanisms [55], [56], [57]. Evidence from recent literature suggests interventions to support parenting offer much scope for improving mental health among children and adolescents later in life [58], [59], [60], [61], [62]. Evidence suggests that early childhood development (ECD) interventions including stimulation in early childhood, preschool level interventions, and ECD consultations have shown to be effective in improving health behaviors, conduct problems, and social skills and are also low-cost interventions delivered in home and at school [63], [64], [65], [66], [67]. Evidence also suggests that ECD and parenting interventions can be implemented effectively in LMICs' schools and community settings; however, evidence for scaling-up and sustainability of mental health promotion interventions in LMICs needs to be strengthened [68].

There are challenges pertaining to adolescent mental health due to the associated stigma. Furthermore, there are gaps related to monitoring the health behavior of adolescents, even with multicountry surveys, for example, most of the data are gathered among older adolescents. More widespread developmentally appropriate surveys of younger adolescents may help identify key ages for implementing preventive mental health interventions. Most population-based adolescent health surveys are conducted in schools. However, even in HICs, there are adolescents who are not in school and who may face significant health inequities. Furthermore, there is little consensus on which indicators and protective factors are the best survey measures. Findings from our review highlight that the existing evidence on mental health interventions for adolescents comes mainly from HICs. There is lack of standardized interventions and outcomes due to which meta-analysis could not be conducted in most of the included systematic reviews. Long-term follow-up data were not available since most of the studies reported outcomes at short-term follow-up, and hence the extent to which the effects of programs were maintained over longer periods of time could not be evaluated. There is a dire need for rigorous, high-quality evidence especially from LMICs on effective interventions to prevent and manage mental health disorders among adolescents. Future trials should also focus on standardized interventions and outcomes for synthesizing the exiting body of knowledge. There is a need to report differential effects for gender, age groups, socioeconomic status, and geographic settings since the impact of mental health interventions might vary according to various contextual factors. Availability of such data would help investigate if certain strategies are more beneficial for one group over the other and developing targeted strategies for various subgroups to optimize effectiveness of interventions.

## Figures and Tables

**Table 1:** Inclusion/exclusion criteria

Inclusion criteria	Exclusion criteria
Systematic review and/or meta-analysis of interventions for prevention and treatment of mental health targeting adolescents (11–19 years) or youth (15–24 years): Eating disorders Anxiety Depression Suicidal behaviors eHealth interventions focusing on adolescent/youth mental health	Nonsystematic reviews Systematic reviews focusing on preventive and therapeutic mental health interventions targeting population other than adolescents and youth Reviews not reporting outcomes related to mental health

**Table 2:** Characteristics of the included reviews

Intervention	Review	Intervention details	Setting; HICs/LMICs	Number of included studies	AMSTAR rating	Outcomes reported
School-based interventions	O'Mara and Lind [18]	Social and emotional health and well-being, positive youth development, health promotion, mental health promotion, primary prevention	Mostly HICs	15 reviews	—	Subclinical internalizing and externalizing problems, academic achievement, mood disorders, anxiety, depressive symptoms, self-concept, self-esteem, coping skills, interpersonal skills, quality of peer and adult relationships, self-control, problem-solving, self-efficacy, school misbehavior, aggressive behavior and violence, interpersonal sensitivity, conflict resolution, school attendance, social functioning
	Mason-Jones et al. [19]	School-based health care including comprehensive services based at schools, dedicated adolescent health services, school-linked services based at local health centers, and servicing a number of schools and other outreach	HICs	27 (RCTs and observational studies)	7	Utilization of mental health services, ever considered suicide, attempted suicide
	Cheney et al. [20]	Nurture group (NG) intervention delivered in primary and secondary school settings. NG sessions typically include circle time meet and greet. A directed activity, aiming to develop cooperation, listening, teamwork, turn-taking, problem-solving, and self-esteem. Snack time. Free time to choose an activity from the range offered. Saying good-byes	HICs	16 (RCTs and pre–post)	8	Social and emotional well-being
	Kim and Franklin [21]	Solution-focused brief therapy on behavioral problems in schools	HICs	7 (RCT, quasi, and case report)	6	Changes in scores from Hare Self-Esteem Scale; Conners' Teacher Rating Scale; Conners' Parent Rating Scale; Feelings, Attitudes and Behaviors Scale for Children; Substance Abuse Subtle Screening Inventory Adolescent-2; and Child Behavior Checklist-Youth.
	Fothergill et al. [22]	Screening tools being used by school nurses for the identification of emotional, psychological, and behavioral problems among adolescents in schools.	HICs	None	6	Existing screening tools being applied by school nurses to detect mental ill health
	Calear and Christensen [23]	School-based prevention and early intervention programs for depression. Mostly including cognitive behavioral therapy (CBT) delivered by a mental health professional or graduate student over 8–12 sessions. Other common therapeutic approaches employed included psychoeducation and interpersonal therapy	HICs	42 RCTs	7	Depression
	Kavanagh et al. [24]	Cognitive behavioral therapy	HICs	17 RCTs	8	Outcome related to depression, anxiety, and suicidality (actual or attempted suicide and suicidal ideation)
	Farahmand et al. [25]	Day therapy programs: a multidisciplinary community-based approach to the treatment of mental health issues	HICs	29 programs	7	Academic outcomes, behavioral outcomes, conduct problems, depression, substance use, internalizing symptoms
	Katz et al. [26]	School-based suicide prevention programs: awareness/education curriculum, gatekeeper training, peer leadership training, screening, skills training, reconnecting youth, good behavior game	HICs	16 programs	5	Students' and school staffs' knowledge and attitudes toward suicide, suicide attempts
	De Silva et al. [27]	Psychological interventions for suicide and self-harm prevention	HICs	38 controlled studies and 6 systematic reviews	6	Mapping of existing literature
	Harrod et al. [28]	Any intervention that (1) targeted students without known suicidal risk (i.e., primary prevention); (2) had the prevention of suicide as one of its primary purposes; and (3) was delivered in the postsecondary educational setting in any country	HICs	8 RCTs	11	Completed suicide, suicide attempt, suicidal ideation, changes in knowledge, attitudes and behaviors
	Harlow and Clough [29]	Suicide prevention programs that have been evaluated for indigenous youth	HICs	11 programs	6	Suicide ideation, knowledge, attitude
Community-based interventions	Bungay and Vella-Burrows [30]	Music, dance, singing, drama and visual arts, taking place in community settings or as extracurricular activities	Mostly HIC except one in Tanzania	20 (RCTs and observational)	5	Behavioral changes, self-confidence, self-esteem, levels of knowledge, and physical activity
	Waddell et al. [31]	Parent training or child social skills training and universal cognitive behavioral therapy (CBT)	HICs	15 RCTs	6	Conduct disorder, anxiety, and depression
	Durlak and Wells [32]	Primary prevention intervention designed specifically to reduce the future incidence of adjustment problems in currently normal populations, including efforts directed at the promotion of mental health	HICs	144 programs	5	Competencies, performance, successful transitions
	Farahmand et al. [33]	Community-based mental health and behavioral programs	HICs	33 (RCTs and observational)	4	Psychological, behavior, achievement, school connectedness, antisocial behavior, interpersonal, social skills community or prosocial activities, physical health
	Bower et al. [34]	Effectiveness of interventions for child and adolescent mental health problems in primary care, and interventions designed to improve the skills of primary care staff	HICs	RCTs and pre–post studies	7	Clinical outcomes, social, educational, satisfaction with treatment, costs, attitudes, knowledge, diagnostic and treatment behavior, costs
Digital platforms	Clement et al. [35]	It was a mass media intervention, defined as an intervention that uses a channel of communication intended to reach large numbers, and is not dependent on person-to-person contact, for example, newspapers, billboards, pamphlets, DVDs, television, radio, cinema, some Web- and mobile phone–based media, street art, and ambient media	HICs	22 RCTs	11	Discrimination or prejudice outcome measures
	Musiat and Tarrier [36]	Computerized cognitive behavioral therapy (cCBT) interventions	HICs	101 (observational studies)	4	Cost-effectiveness, geographic flexibility, time flexibility, waiting time for treatment, stigma, therapist time, effects on help-seeking and treatment satisfaction
	Montgomery et al. [27]	Media-based cognitive behavioral therapies	HICs	11 RCTs	11	Behavioral disorders, therapist time
	Clarke et al. [38]	Online mental health promotion and prevention interventions	HICs	28 observational studies	6	Anxiety, depression
	Calear and Christensen 2010 [39]	BRAVE for Children—ONLINE and BRAVE for Teenagers—ONLINE: based on cognitive behavioral therapy (CBT), these programs consist of 10 weekly sessions for children and adolescents; two booster sessions presented 1 and 3 months after the intervention, and five or six parent sessions. The programs present information on managing anxiety, recognizing the physiological symptoms of anxiety, graded exposure, and problem-solving techniques. Project CATCH-IT is a free, Internet-based training program based on behavioral activation, CBT, and interpersonal psychotherapy. MoodGYM is a free, interactive, Internet-based program designed to prevent and decrease symptoms of depression in young people. Grip op je dip online is a free, Dutch language, CBT-based program aimed at 16- to 25-year-olds. Based on the face-to-face Grip op je dip course, the online program consists of six moderated chat sessions attended by six to eight participants.	HICs	4 programs	9	Anxiety and depression
	Kauer et al. [40]	Online services in facilitating mental health help-seeking	HICs	18 (RCTs and observational studies)	9	Help-seeking, mental health
	Martin et al. [41]	Networked communication: e-mail and/or Web-based electronic diary; videoconference; and virtual reality.	HICs	12 (RCTs and observational studies)	9	Clinical outcomes (e.g., symptom alleviation), patient-level impacts (e.g., improved health behaviors), patient and health care professional satisfaction and costs
	Farrer et al. [42]	A range of broad technology types including the Internet, audio, virtual reality, video, stand-alone computer programs, and/or a combination of these	HICs	27 RCTs	9	Depression, anxiety
Individual-/family-based interventions	Pratt and Woolfenden [43]	Eating disorder awareness, promotion of healthy eating attitudes and behaviors, as well as eating disorder awareness and coping with general adolescent issues, training in media literacy and advocacy skills	HIC	12 RCTs	8	BMI, Eating Attitude Test, Eating Disorder Inventory, Sociocultural Attitudes Towards Appearance Questionnaire, social perception profile, body image assessment
	Ekelend et al. [44]	Gross motor, energetic activity, for example, running, swimming, ball games and outdoor play of moderate to high intensity, or strength training, in contrast to “ordinary” physical activity (e.g., routine physical education (PE) classes, walking to school, or playtime activities of low intensity) for at least a duration of 4 weeks	Mostly HIC except one in Nigeria	23 RCTs	8	Self-esteem
	Lubans et al. [45]	Three types of physical activity programs (i.e., outdoor adventure, sport and skill-based and physical fitness programs)	HICs	15 (RCTs, quasi, and pre–post)	9	Social and emotional well-being
	Cooney et al. [46]	Exercise was defined as “planned, structured and repetitive bodily movement done to improve or maintain one or more components of physical fitness”	Mostly HICs except one in Thailand, one in Brazil	39 RCTs	11	Depression, acceptability of treatment, number of participants completing the interventions; quality of life; cost; adverse events
	Larun et al. [47]	Interventions that included vigorous physical activity of clearly specified quality with a minimum duration of 4 weeks	HICs	16 RCTs	11	Anxiety or depression symptoms post-treatment
	James et al. [48]	(1) The relative efficacy of CBT versus non-CBT active treatments; (2) the relative efficacy of CBT versus medication and the combination of CBT and medication versus placebo; and (3) the long-term effects of CBT	HICs	41 RCTs	11	Remission, reduction in anxiety symptom, acceptability
	Cox et al. [49]	Any psychological therapy with any antidepressant medication; a combination of interventions (psychological therapy plus antidepressant medication) with either psychological therapies or antidepressant medication alone; a combination of interventions (psychological therapy plus antidepressant medication) compared with either intervention (psychological therapy or antidepressants) plus a placebo; and a combination of interventions (psychological therapy plus antidepressant medication) with a placebo or treatment as usual	HICs	11 RCTs		Remission from depressive disorder, acceptability, suicide-related serious adverse events, dropouts
	Cox et al. [50]	Any type of pharmacotherapy or psychological therapy	HICs	9 RCTs	11	Prevention of a second or next episode, readmissions, time to relapse, functioning, depressive symptoms, dropouts, secondary morbidity
	Shinohara et al. [51]	Behavioral therapy, behavioral activation, social skills training assertiveness training, relaxation therapies, other psychological therapies	HICs	25 RCTs	11	Treatment efficacy, treatment acceptability, remittance, improvement in depressive symptoms, improvement in other symptoms
	Weisz et al. [52]	Evidence-based psychotherapies	HICs	52 RCTs	8	Measures of symptoms and functioning
	Shepperd et al. [53]	Mental health services providing specialist care, beyond the capacity of generic outpatient provision, which provide an alternative to inpatient mental health care	HICs	7 RCTs	11	Disease-specific symptoms, general psychological functioning, acceptability, and cost
	Deenadayalan et al. [54]		HICs	8 RCTs and observational studies	6	Symptoms, knowledge, attitude

AMSTAR = assessment of the methodological quality of systematic reviews criteria; BMI = body mass index; HIC = high-income country; LMIC = low- and middle-income country; RCT = randomized controlled trial.

**Table 3:** Summary estimates for adolescent mental health interventions

Interventions (number of reviews)	Comparison	Outcomes and estimates
School-based interventions (n = 12)	School-based CBT	Symptoms of depression: effect size range: .21 to 1.40
	CBT in secondary schools	Depression **(SMD: −.16; 95% CI: −.26 to −.05)**
		Anxiety **(SMD: −.33; 95% CI: −.59 to −.06)**
	Classroom instructions	Knowledge of suicide **(SMD: 1.51; 95% CI: .57 to 2.45)**
		Knowledge of suicide prevention **(SMD: .72; 95% CI: .36 to 1.07)**
Community-based interventions (n = 6)	Person-centered programs	Social acceptance at 3-month follow-up *(SMD: −.03; 95% CI: −.10 to .04)*
		Affective education **(SMD: .33; 95% CI: .18 to .48)**
		Aggregate of positive mental health outcome *(SMD: .03; 95% CI: −.19 to .25)*
	Person plus environment interventions	Aggregate of positive mental health outcome **(SMD: .27; 95% CI: .16 to .37)**
	Environment-only interventions	Aggregate of positive mental health outcome **(SMD: .38; 95% CI: .15 to .60)**
Digital platforms (n = 8)	Mass media	Discrimination: effect size range: SMD −.85 to −.17
		Prejudice: effect size range: SMD −2.94 to 2.40
Individual-/family-based interventions (n = 12)	Media literacy and advocacy approach	Internalization or acceptance of societal ideals relating to appearance at a 3- to 6-month follow-up **(SMD: −.28; 95% CI: −.51 to −.05)**
	Eating attitudes and behaviors and adolescent issues	BMI at 12- to 14-month follow-up *(SMD: −.10; 95% CI: −.45 to .25)*
		Eating Attitude Test at 6- to 12-month follow-up *(SMD: .01; 95% CI: −.13 to .15)*
		Eating Disorder Inventory “bulimia” at 12- to 14-month follow-up *(SMD: −.03; 95% CI: −.16 to .10)*
	Self-esteem approach	Close friendship at 3-month follow-up *(SMD: −.01; 95% CI: −.09 to .06)*
	Exercise alone	Self-esteem **(SMD: .49; 95% CI: .16 to .81)**
	Exercise as a part of a comprehensive intervention	Self-esteem **(SMD: .51; 95% CI: .15 to .88)**
	Exercise compared to control	Depression **(SMD: −.62; 95% CI: −.81 to −.42)**
		Dropouts *(RR: 1.00; 95% CI: .97 to 1.04)*
	Exercise compared to psychological therapies	Depression *(SMD: −.03; 95%CI −.32 to .26)*
	Exercise compared to antidepressant	Depression *(SMD: −.11; 95% CI: −.34 to .12)*
	Vigorous exercise versus no intervention	Anxiety scores *(SMD: −.48; 95% CI: −.97 to .01)*
		Depression score **(SMD: −.66; 95% CI: −1.25 to −.08)**
	Vigorous exercise to low intensity exercise	Anxiety scores *(SMD: −.14; 95% CI: −.41 to .13)*
		Depression scores *(SMD: −.15; 95% CI: −.44 to .14)*
	Exercise with psychosocial interventions	Anxiety scores *(SMD: −.13; 95% CI: −.43 to .17)*
		Depression scores *(SMD: .10; 95% CI: −.21 to .41)*
	Waitlist versus CBT for anxiety	Anxiety remission **(OR: 7.85; 95% CI: 5.31 to 11.6)**
		Participants lost to follow-up: *(OR: .93; 95% CI: .58 to 1.51)*
	Psychological therapy versus antidepressant medications for depression	Remission *(OR: .62; 95% CI: .28 to 1.35)*
		Dropouts *(OR: .61; 95% CI: .11 to 3.28)*
		Suicidal ideation **(SMD: −3.12; 95% CI: −5.91 to −.33)**
		Depression symptoms *(SMD: .16; 95% CI: −.69 to 1.01)*
	Combination therapy versus antidepressant medication for depression	Remission *(OR: 1.50; 95% CI: .99 to 2.27)*
		Dropouts *(OR: .84; 95% CI: .51 to 1.39)*
		Suicidal ideation *(OR: .75; 95% CI: .26 to 2.16)*
		Depression symptoms *(SMD: −.27; 95% CI: −4.95 to 4.41)*
		Functioning *(SMD: .09; 95% CI: −.11 to .28)*
	Combination therapy versus psychological therapy	Remission *(OR: 1.61; 95% CI: .38 to 6.90)*
		Dropouts *(OR: 1.23; 95% CI: .12 to 12.71)*
		Suicidal ideation *(SMD: .60; 95% CI: −2.25 to 3.45)*
		Depression symptoms *(SMD: −.28; 95% CI: −1.41 to .84)*
	Combination therapy versus psychological therapy plus placebo	Dropouts *(OR: .98; 95% CI: .42 to 2.28)*
		Remission **(OR: 2.15; 95% CI: 1.15 to 4.02)**
		Depression symptoms **(SMD: −.52; 95% CI: −.78 to −.26)**
	Antidepressants compared to placebo to relapse and recurrence	Number of relapsed recurred **(OR: .34; 95% CI: .18 to .64)**
		Suicide-related behaviors *(OR: 1.02; 95% CI: .14 to 7.39)*
		Dropouts *(OR: 1.02; 95% CI: .38 to 2.79)*
	Behavioral therapy compared to all other psychological therapies	Response *(RR: .97; 95% CI: .86 to 1.09)*
		Remission *(RR: .91; 95% CI: .8 to 1.04)*
		Response at follow-up *(RR: .77; 95% CI: .59 to 1.01)*
		Depression severity *(SMD: −.03; 95% CI: −.2 to .15)*
		Dropouts *(RR: 1.02; 95% CI: .65 to 1.61)*
	Evidence-based youth-focused psychotherapy versus usual clinical care	Effect size **(SMD: .31; 95% CI: .16 to .44)**
	Evidence-based parent-/family-focused psychotherapy versus usual clinical care	Effect size *(SMD: .16; 95% CI: −.01 to .33)*
	Multisystem approaches	Effect size *(SMD: .35; 95% CI: .19 to .52)*
	Combinations	Effect size *(SMD: .29; 95% CI: .06 to .52)*

Bold indicates significant impact. Italics indicate nonsignificant impact.

BMI = body mass index; CBT, cognitive behavioral therapy; CI = confidence interval; OR = odds ratio; RR = relative risk; SMD = standard mean difference.
